# Atypical presentation of occupational mpox: a case report from Brazil

**DOI:** 10.1128/asmcr.00104-24

**Published:** 2025-08-01

**Authors:** Guilherme S. Lira, Mariana Q. S. Melo, Isabela C. Leitão, Matheus V. Andrade, Anna C. Castiñeiras, Diana Mariani, Cássia C. A. Gonçalves, Lendel C. Costa, Danielle A. S. Rodrigues, André M. Vale, Filipe R. R. Moreira, Carolina M. Voloch, Débora S. Faffe, Luiza M. Higa, Rafael M. Galliez, Clarissa R. Damaso, Amilcar Tanuri, Terezinha M. P. P. Castiñeiras

**Affiliations:** Rush University Medical Center, Chicago, Illinois, USA; 1 Núcleo de Enfrentamento e Estudos de Doenças Infecciosas Emergentes e Reemergentes, Universidade Federal do Rio de Janeiro 89111, Rio de Janeiro, Brazil; 2 Faculdade de Medicina, Universidade Federal do Rio de Janeiro, Rio de Janeiro, Brazil; 3 Laboratório de Virologia Molecular, Departamento de Genética, Instituto de Biologia, Universidade Federal do Rio de Janeiro 89111, Rio de Janeiro, Brazil; 4 Instituto de Biofísica Carlos Chagas Filho, Universidade Federal do Rio de Janeiro 28125, Rio de Janeiro, Brazil

**Keywords:** mpox, occupational, outbreak, healthcare worker, case report

## Abstract

**Background:**

After decades of circulating predominantly in Africa, mpox caused a public health emergency of international concern between 2022 and 2023. Even outside of the epidemic period, the virus still circulates, with threats of reemergence, and different clinical presentations must be brought to attention for optimal surveillance.

**Case Summary:**

We report a case of monkeypox virus (MPXV) infection in a previously healthy physician with an atypical clinical presentation and a single identified exposure. Diagnosis was confirmed by PCR. Viral isolation was employed to reinforce diagnosis. Plaque reduction neutralization assays and semiquantitative ELISA were used to evaluate the antibody response.

**Conclusion:**

Our report has two main messages: atypical symptoms of mpox can hinder diagnosis, making adequate suspicion and testing fundamental; and the risk of occupational exposure must be considered when designing policies for healthcare workers.

## INTRODUCTION

The World Health Organization (WHO) declared mpox a public health emergency of international concern due to a multicountry outbreak related to clade IIb transmission from July 2022 to May 2023 (1). Brazil recorded more than 10,000 cases and the second-highest number of deaths (2). Though cases have significantly waned, mpox has not been eliminated. Of note, since 2024, an mpox outbreak related to clade I virus has been affecting the Democratic Republic of the Congo (DRC) with more than 330,000 suspected cases and 1,000 deaths. The current outbreak has spread to neighboring countries, and there have also been travel-associated cases outside Africa (3), which is concerning due to potentially worse outcomes associated with this clade. In the 2022–2023 outbreak, sexual exposure was a common feature (4, 5). However, respiratory droplets, contact surfaces, and infected skin lesions are also known means of transmission of monkeypox virus (MPXV) (6), accounting for the occurrence of family cluster cases since the 1970s. Mpox cases were reported in healthcare workers (HCWs), but most resulted from community exposure, few were genuinely occupational (7), and mostly happened through the percutaneous route with needlestick injuries (8). Here, we report the case of a physician with a confirmed mpox infection probably acquired through occupational exposure, with atypical lesions and no systemic symptoms.

## CASE PRESENTATION

**Fig 1:**
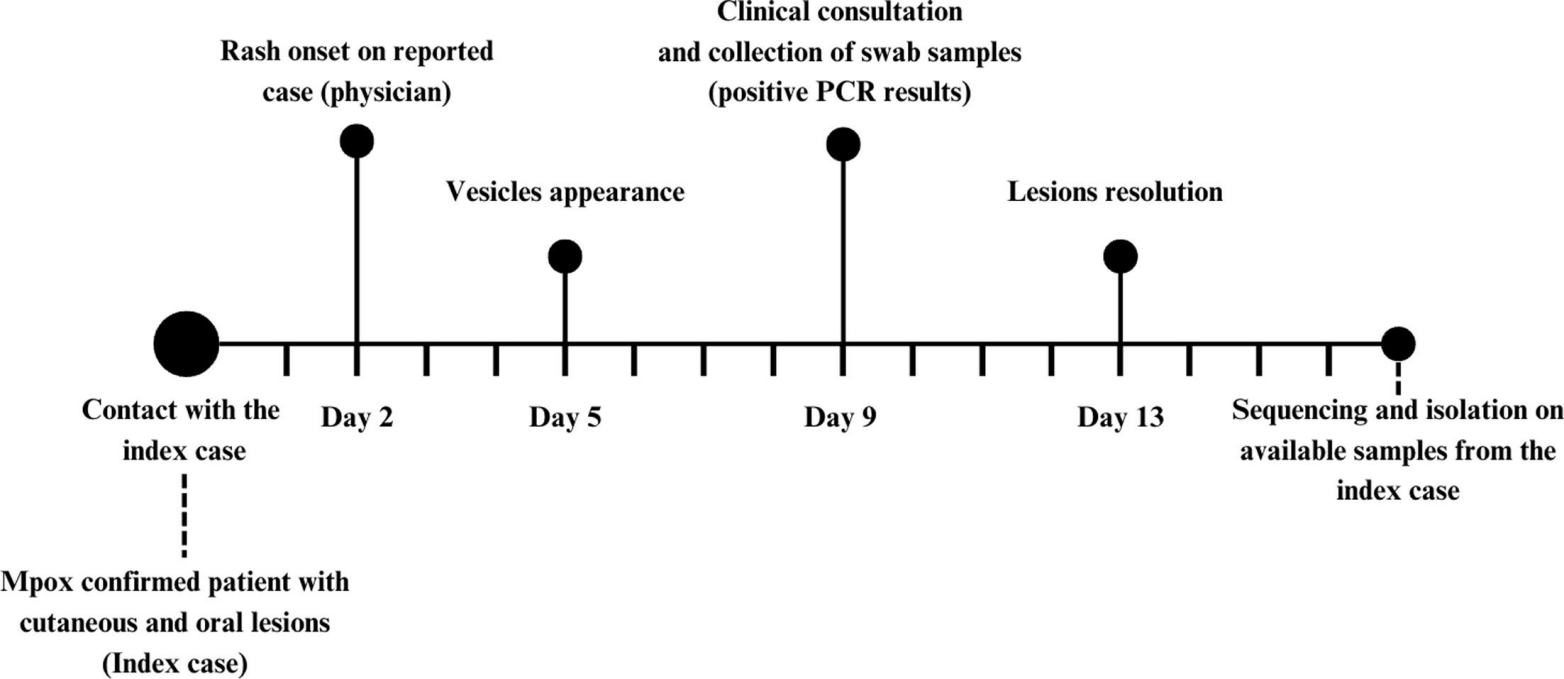
Historical information from the occupational mpox case organized as a timeline.

The reported case is a 62-year-old heterosexual male, previously healthy, working as a physician. He reported a 7-day history of acute, pruritic skin eruption, initially characterized by hives on the waistline in February 2024. Considering his previous medical history of atopy, the eruption was self-attributed to fabric softeners. Three days after the waistline rash onset, small vesicles appeared on the anterior pelvis, buttocks, and limbs. Considering the possibility of an allergic reaction with a secondary cutaneous bacterial infection, the physician self-prescribed loratadine and amoxicillin-clavulanate, without symptom improvement. On his seventh day of illness, concerned about a recent contact with an mpox-suspected case in his private clinic, he presented for a medical consultation in the reference center for mpox diagnosis of the Federal University of Rio de Janeiro (Fig. 1).

Approximately 60 hours preceding his rash onset, he had attended an HIV-positive patient in his private clinic with a nonspecific complaint of malaise. He reported greeting his patient with a handshake upon arrival, washing his hands, and performing the consultation without personal protection equipment (PPE). Once the patient complained of cutaneous lesions, he inched closer and noticed vesicles, realizing it could be mpox. He rewashed his hands and maintained a distance of 2 meters between himself and the patient for the rest of the appointment, which lasted an estimated 15 minutes. Besides this patient, he denied other epidemiological links to probable or confirmed mpox cases in the previous four weeks, such as unprotected sexual exposure or contact with other mpox cases, as he was on vacation and made an exception to attend this patient. Of note, he had been vaccinated for smallpox as a child.

**Fig 2:**
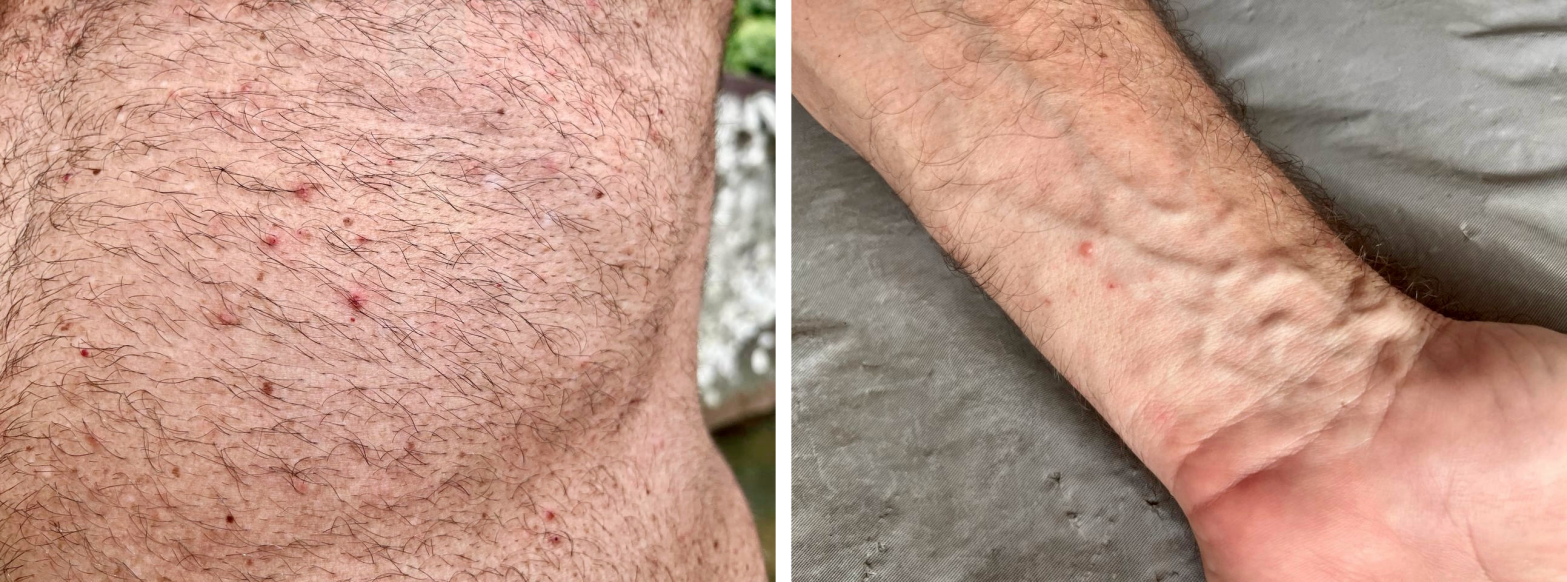
Vesicular and crusted lesions on the abdomen (left panel) and the forearm (right panel) observed in the physician.

During the physician’s consultation in our center, he denied any other symptoms, including fever, malaise, lymphadenopathy, and throat soreness, even in the two weeks preceding rash onset. In the physical examination, twenty vesicles measuring around 3 mm in diameter were observed, distributed on the forearms, thighs, chest, and buttocks, most on erythematous skin (Fig. 2). Some crusted lesions were also present on the buttocks. We collected swab samples from vesicle fluid of forearm and thigh lesions, humid crusts from his buttocks, and a blood sample. Mpox diagnosis was investigated through real-time PCR of clinical samples with a commercial kit (Bio-Manguinhos Kit Molecular multiplex OPXV/ MPXV/VZV/RP, Rio de Janeiro, Brazil) (9). All cutaneous samples yielded positive MPXV-PCR results, with cycle thresholds (Cts) between 29 and 31, confirming mpox diagnosis. Blood samples yielded negative MPXV-PCR results. He was advised to remain in isolation until all lesions were re-epithelialized. Antiviral therapy and anti-orthopoxvirus vaccination were not promptly available and were not employed. Lesions resolved in five days after sample collection with no further complications.

**Fig 3:**
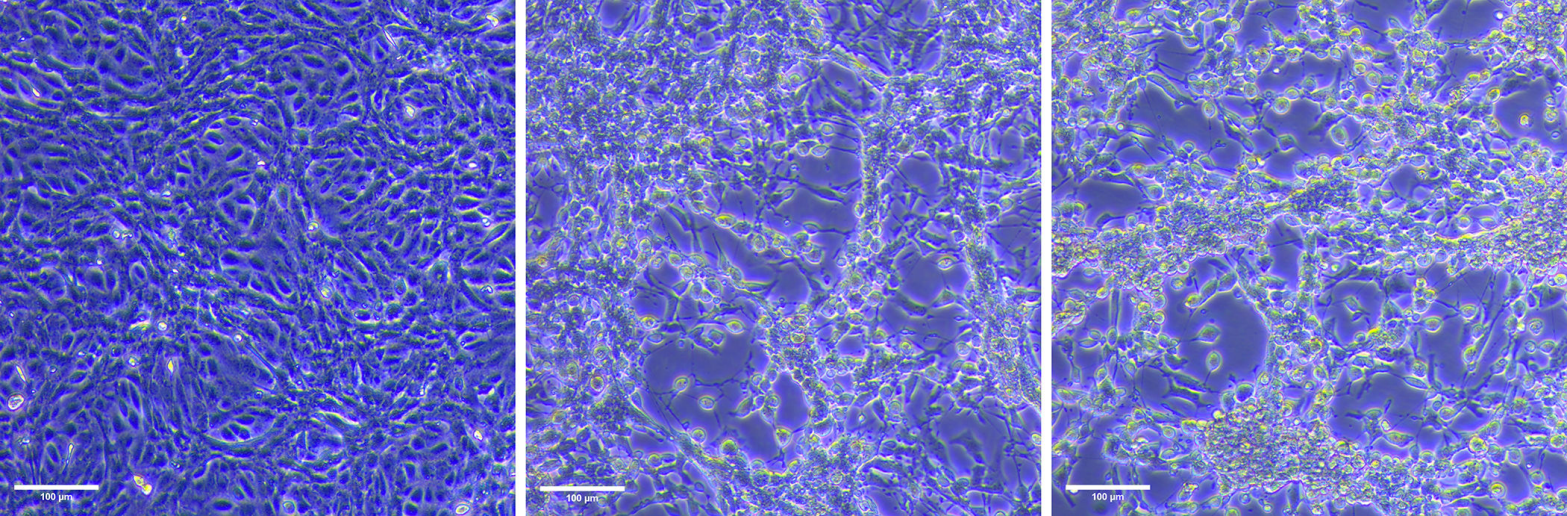
BSC-40 cells (left, uninfected) exhibited cytopathic effect three days after inoculation of oropharyngeal swab eluate (middle) and cutaneous lesion swab eluate (right) from the patient (presumable index case). 100 µm magnification bars are indicated at the bottom left.

In an effort to unequivocally confirm occupational transmission, we attempted to perform viral isolation and sequencing on all available samples related to the case, i.e., from the physician positive cutaneous samples and from his patient (index case), who provided diverse samples, all positive, including an oropharyngeal swab (Ct = 21), three cutaneous lesion swabs (Cts between 18 and 25), and serum (Ct = 32). Virus from the oropharyngeal and cutaneous samples was isolated in BSC-40 cells (Fig. 3). Accordingly, culture supernatants of these swab samples were positive for MPXV. We sequenced the MPXV genome of the original patient’s samples and found that it belonged to clade IIb, lineage B.1.22 (publicly available on GISAID, EPI_ISL_19140077). Numerous attempts at viral isolation and sequencing in a positive serum sample from the patient (Ct = 32) and on all the physician’s positive samples (Cts between 29 and 31) were unsuccessful.

**Fig 4:**
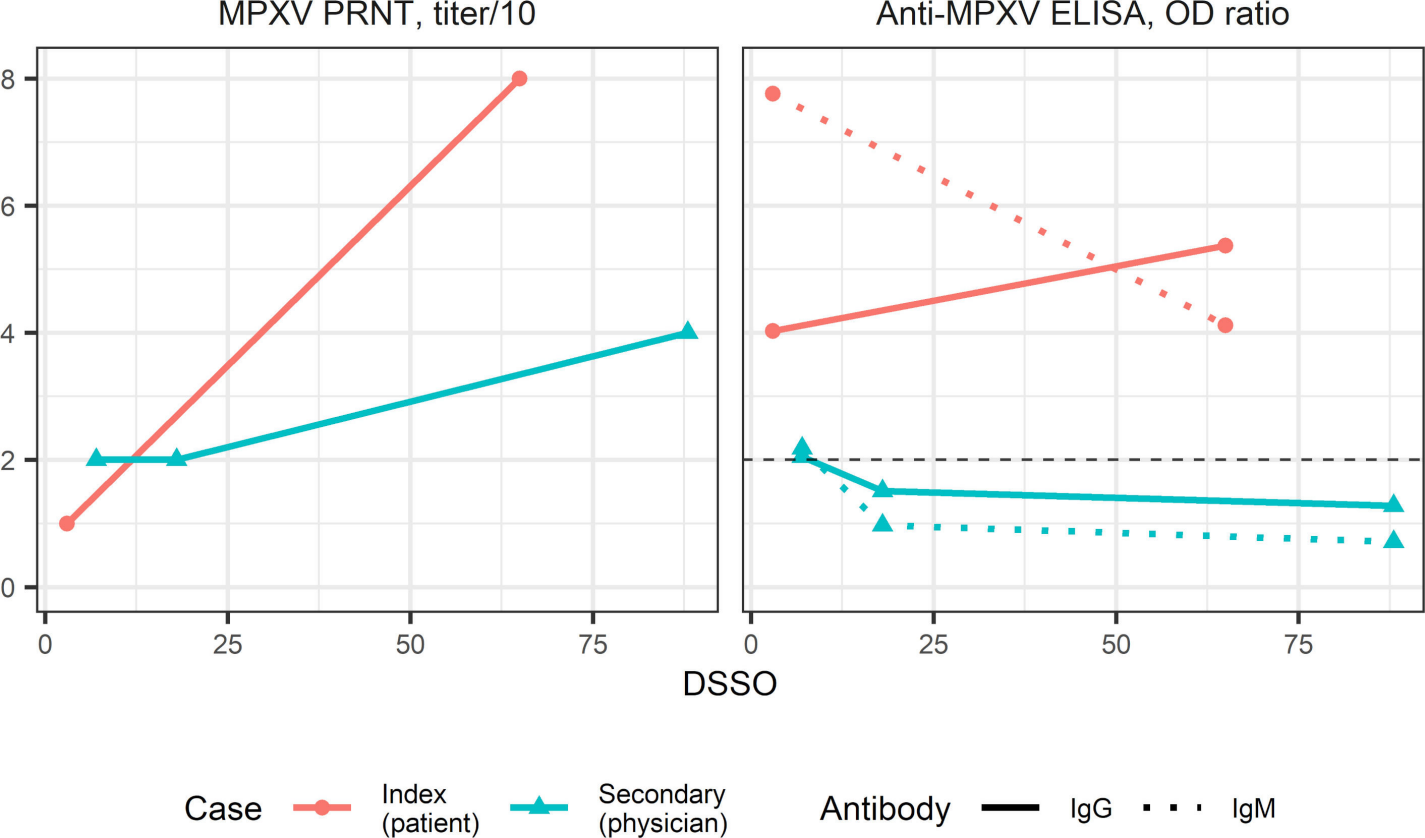
Antibody responses evaluated by MPXV PRNT and anti-MPXV ELISA. Red circles represent serum samples from the index case. Blue triangles represent serum samples from the physician. Solid lines connect IgG results, while dotted lines indicate IgM detection assays. Dashed horizontal line in the ELISA panel indicates the threshold of positivity. The physician showed only a twofold increase in NAbs, of questionable significance. The index patient had detectable and increasing levels of anti-MPXV antibodies. His production of neutralizing antibodies also increased fourfold.

To explore aspects of host immune response in this case, we also evaluated antibody responses through plaque reduction neutralization tests (PRNT) and enzyme-linked immunosorbent assays (ELISA) based on orthopoxvirus antigens (Fig. 4). The physician showed only a minimal, twofold PRNT titer variation between the convalescent phase to 3 months after symptom onset, which does not constitute evidence of increasing neutralizing capability. The index patient had detectable and increasing levels of anti-MPXV antibodies. His production of neutralizing antibodies also increased, from being detected at 1:10 dilution to 1:80 dilution (Fig. 3).

## DISCUSSION

As public health efforts shift to a long-term commitment to control measures, sustained surveillance, and research on mpox (1), this case report serves two purposes: first, to highlight atypical features of a case to improve awareness of other unusual cases; and second, to discuss preventive measures against the disease, including during routine patient care.

Classically, mpox was described as a generalized rash, beginning on the face, preceded by systemic symptoms such as fever and lymphadenopathy, progressing simultaneously through maculopapular, vesicular, pustular, and crust formation stages, and more commonly seen in children (10). The 2022–2023 mpox outbreak was notable for distinct clinical presentations—sometimes without systemic symptoms, frequent anogenital involvement, and simultaneous lesions at different stages—and cases mostly among men who have sex with men (11).

Occupational exposure to MPXV among healthcare and laboratory workers cannot be overlooked and has led to infections in previous outbreaks. Accidents with contaminated materials can lead to disease through the percutaneous route, usually localized and featuring only a puncture-site wound (12). In contrast, infections through other routes of exposure frequently yield systemic symptoms. In September 2018, a transmission case from a patient with a travel-associated infection to a HCW was reported in the United Kingdom. The only exposure risk identified in the case was the changing of potentially contaminated bedding of the patient wearing only a gown and gloves but not eye protection and an N95 mask (13).

To our knowledge, this is the first report of a probable occupational mpox case acquired most likely through the respiratory or by contact route with no systemic symptoms. Moreover, the atypical presentation delayed suspicion even by a trained physician, who first suspected allergic and bacterial etiologies. The present report underscores the well-known necessity of diagnostic tools in investigating infectious diseases, particularly molecular testing. However, we acknowledge the limitation that our report has in establishing accurate transmission linkage due to the lack of successful sequencing from both cases.

Following the recommendations of the Smallpox Eradication Campaign held in Brazil until 1970, the physician presented in our report received the smallpox vaccine in childhood. Due to the vaccine cross-protection, he could have been partially protected against more symptomatic disease (14). Although previous works consistently show smallpox-vaccinated individuals to be at lower risk of mpox (15, 16), the immune response to infection in this population is still poorly characterized, but there are reports of historically vaccinated patients with low antibody titers (17). The weak antibody response seen makes the hypothesis of antibody cross-protection less likely, but innate immunity and cellular immunity might have played a more prominent role.

The use of PPE must also be continuously promoted in all clinical settings. MPXV has demonstrated an ability to circulate through interhuman transmission, including asymptomatic forms, without zoonotic cycles. Thus, new cases are expected even outside of epidemics, and disease continues to circulate worldwide with occasional upsurge in cases and outbreaks, as noted in 2024 (18). The potential spread of the clade Ib epidemic beyond the DRC is of particular concern (3). The disease should thus be carefully considered as a differential diagnosis for general malaise, even before the report of acute skin eruptions, particularly in high-risk populations, and ruled out accordingly. Healthcare providers must be aware of mpox features not only to diagnose, advise, and treat their patients adequately but also to assess their own risk of exposure and implement protective measures.

### Conclusion

Mpox transmission is still occurring worldwide, and HCWs should be aware of transmission routes and prevention measures, including correct use of PPE, which should not be restricted to patients who seek assistance due to skin lesions, but also those complaining of non-specific systemic symptoms, particularly in high-risk populations.
